# Notch2 as a promising prognostic biomarker for oesophageal squamous cell carcinoma

**DOI:** 10.1038/srep25722

**Published:** 2016-05-09

**Authors:** Cong Wang, Qingbao Li, Fang Liu, Xuan Chen, Bowen Liu, Effat Un Nesa, Shanghui Guan, Lihui Han, Bingxu Tan, Nana Wang, Xintong Wang, Qingxu Song, Yibin Jia, Jianbo Wang, Ming Lu, Yufeng Cheng

**Affiliations:** 1 Department of Radiation Oncology, Qilu Hospital of Shandong University, Jinan, Shandong, 250012, China; 2 Department of Cardiac Surgery, Shandong Provincial Hospital Affiliated to Shandong University, Jinan, Shandong, 250021, People’s Republic of China; 3 Department of Imaging, Shandong Medical College, Jinan, Shandong, 250002, China; 4 Department of Radiation Oncology, Shandong Cancer Hospital and Institute, Jinan, Shandong, 250117, China; 5 Department of Thoracic Surgery, Qilu Hospital of Shandong University, Jinan, Shandong, 250012, China

## Abstract

We aimed to examine Notch2 expression in oesophageal squamous cell carcinoma (ESCC) patients and to evaluate its prognostic potential. Immunohistochemical (IHC) staining, quantitative real-time polymerase chain reaction (qRT-PCR) and western blot analysis were utilized to investigate the Notch2 expression status and prognostic value. Furtherly, CCK8 and clonogenic assays were conducted to determine if Notch2 inhibition by shRNA could lead to a decrease in the proliferation and survival of ESCC cells. A notably higher Notch2 expression level was found in ESCC tissues at the mRNA (*P* < 0.0001) and protein levels (IHC: *P* = 0.004; western blot: *P* = 0.021). Log-rank analysis demonstrated that Notch2 overexpression was significantly associated with worse overall survival (OS) (29.1% vs. 49.1%; *P* = 0.013) and progression-free survival (PFS) (15.3% vs. 34.4%; *P* = 0.006) rates in ESCC patients. The multivariate analysis revealed Notch2 as an independent prognostic factor for OS and PFS (*P* = 0.002 and 0.006, resp.). Besides, *in vitro* assays showed that OD450 values and colony formations were significantly reduced in Notch2-shRNA group (all *P* < 0.0001). In conclusion, these results show that Notch2 is up-regulated in ESCC tissues and could serve as a promising biomarker for identifying individuals with poor prognostic potential.

Oesophageal cancer is the sixth leading cause of cancer-related mortality and the eighth most common cancer worldwide^1^. The so-called Asian belt, which encompasses Turkey, northeastern Iran, Kazakhstan, and northern and central China, has a very high incidence of oesophageal squamous cell carcinoma (ESCC). There are more than 100 cases per 100,000 individuals in this population annually, and the incidence is equal in men and women^2^. In the USA, 16,980 new oesophageal cancer cases were diagnosed in 2015 and 15,590 deaths were estimated to occur in the same year^3^. The overall 5-year survival rate of oesophageal cancer patients ranges from 15% to 25%, even with the development of new diagnostic and treatment methods in recent years^4^. Therefore, there is an urgent need to identify novel biomarkers that will provide better prognoses and individualization of treatment.

The Notch pathway has emerged as one of the major signalling cascades activated throughout development, and its misregulation has been associated with many diseases^5^. In total, mammals express four Notch receptors: Notch1, Notch2, Notch3 and Notch4. The dysregulation of Notch2 has been reported in human haematological malignancies and various solid tumours. Indeed, Notch2 mutations play a role in the pathogenesis and progression of splenic marginal zone lymphoma and are associated with poor prognoses^6^. A truncate mutation of Notch2 was shown to enhance cell proliferation by activating the NF-κB signal pathway in diffuse large B-cell lymphoma^7^. Notch2 has also been reported to be overexpressed in lung adenocarcinoma^8^, glioma^9^, cervical cancer^10^,^11^, hepatoblastoma^12^, gastric cancer^13^,^14^ and salivary adenoid cystic carcinoma^15^. By contrast, Notch2 is down-regulated and plays suppressive roles in bladder cancer^16^, lung cancer^17^, breast cancer^18^,^19^ and colorectal cancer^20^. Notably, its expression in ovarian cancer depends on the histological type. Papillary serous, endometrioid and clear cell ovarian cancers show negative or low Notch2 expression, while mucinous carcinomas show a high expression level^21^. As a result, the role of Notch2 in tumourigenesis remains controversial.

Song Y *et al*.^22^ conducted whole-genome sequencing and whole-exome sequencing in ESCC patients and identified significantly mutated genes, including genes involved in Notch signalling. Similarly, Notch2 was reported to be more frequently altered in ESCC compared with oesophageal adenocarcinoma via a comprehensive genomic profiling method^23^,^24^,^25^ and immunohistochemical (IHC) staining^26^. At present, the precise association between Notch2 and ESCC prognosis has yet to be elucidated. The present study used IHC, qRT-PCR and western blot to examine the expression of Notch2 in human ESCC tissues and paracancerous tissues. In addition, the association between Notch2 and various clinicopathological characteristics was investigated, along with the predictive potential of Notch2 in ESCC. Further *in vitro* assays were used to verify its effects in proliferation and survival of ESCC cells.

## Results

### Notch2 expression in frozen ESCC tissues

**Figure 1:**
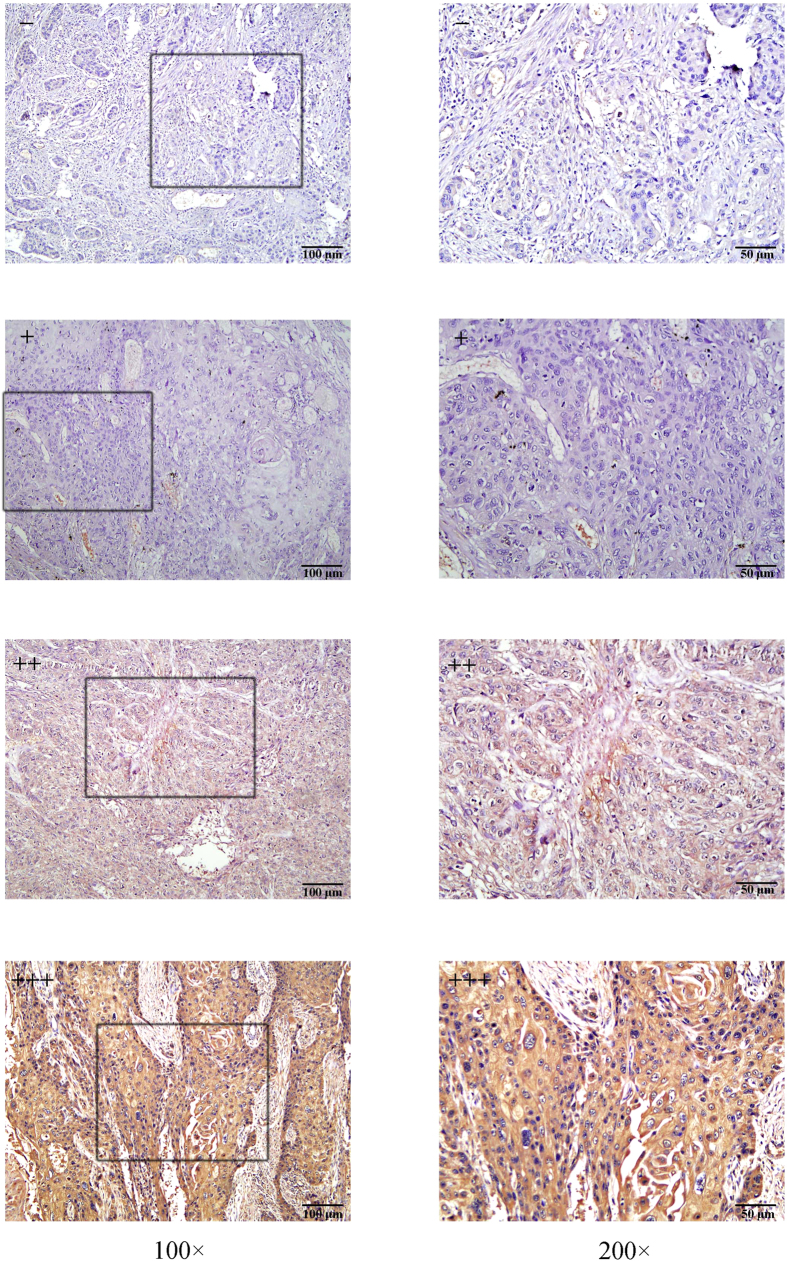
Immunohistochemical staining of Notch2 in ESCC tissues, which were graded as (−), (+), (++), or (+++) (100× and 200×, resp.). Notch2 expression was primarily localized to the cytoplasm of cancer cells.

**Figure 2:**
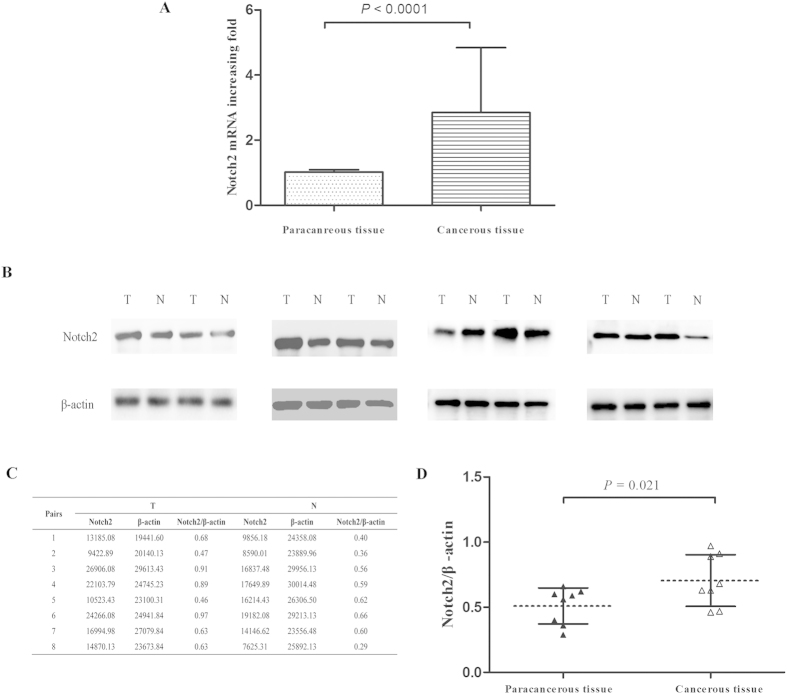
Notch2 mRNA and protein expression levels in ESCC tissues compared with matched paracancerous tissues. (**A**) Notch2 mRNA expression in ESCC tissues compared with matched paracancerous tissues via qRT-PCR. The bands (**B**) and absolute intensity values (**C**) of Notch2 and β-actin in 8 pairs of ESCC and matched paracancerous tissue samples via western blot analysis. (**D**) Quantitative analysis of Notch2 protein expression in ESCC and matched paracancerous tissues normalized to β-actin expression. Abbreviations: T, cancerous tissues; N, paracancerous tissues. The results are expressed as the mean ± standard deviation.

We investigated the Notch2 mRNA and protein expression levels in 30 paired cancerous tissues and matched paracancerous tissues using qRT-PCR, IHC and western blot analysis. As shown in Fig. 1, Notch2 expression was primarily localized to the cytoplasm of cancer cells. IHC staining revealed that Notch2 was overexpressed in 63.3% (19/30) of cancerous tissues and 26.7% (8/30) of paracancerous tissues, and the difference between these levels was statistically significant (*P* = 0.004, Table 1). The qRT-PCR analysis revealed that Notch2 was up-regulated in cancerous tissues compared with matched paracancerous tissues in 19 (63.3%) cases (*P* < 0.0001). The mean fold increase in Notch2 mRNA in cancerous tissues was 4.71 ± 1.19 vs 1.04 ± 0.11 (Fig. 2A). Furthermore, we randomly selected 8 pairs of cancerous and paracancerous tissues to identify the Notch2 protein level using western blot analysis (Fig. 2B). The results showed higher Notch2 protein expression in cancerous tissues than in adjacent tissues (Notch2/β-actin: 0.70 ± 0.20 vs. 0.51 ± 0.14, *P* = 0.021, Fig. 2C,D).

### The prognostic value of Notch2 in ESCC

**Figure 3:**
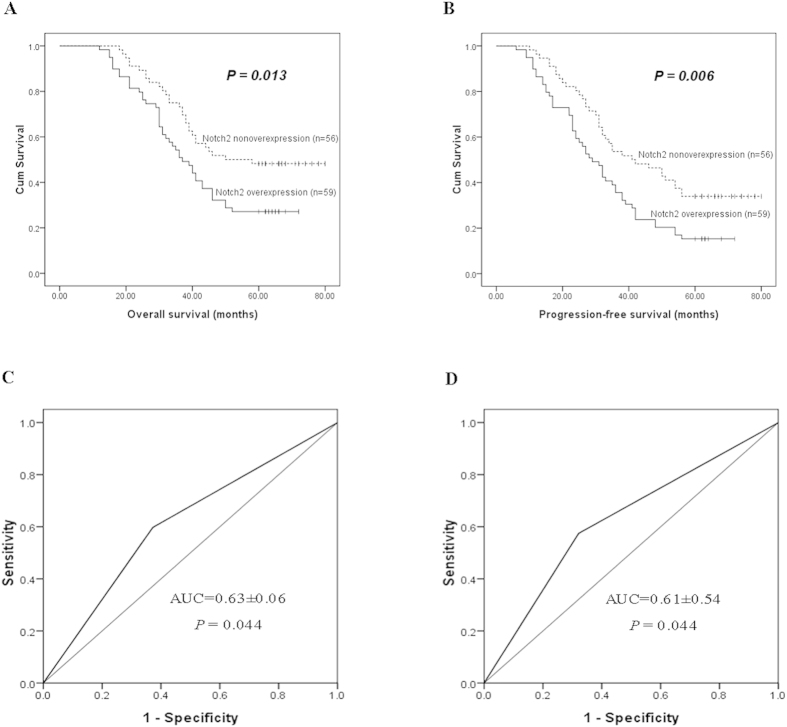
(**A**) Kaplan-Meier analysis and log-rank test of Notch2 for OS. High Notch2 protein expression significantly predicted decreased OS. (**B**) Kaplan-Meier analysis and log-rank test of Notch2 for PFS. High Notch2 protein expression was significantly associated with decreased PFS. ROC curve for Notch2 according to OS (**C**) and PFS (**D**) predictions. The AUC value was 0.63 ± 0.06 (*P* = 0.044) according to the OS prediction and 0.61 ± 0.54 (*P* = 0.044) according to the PFS prediction. Abbreviations: OS: overall survival; PFS: progression-free survival.

Of the 115 patients who provided formalin-fixed paraffin-embedded (FFPE) cancer tissues, 43 (37.4%) survived more than 5 years after subtotal esophagectomy and 72 (62.6%) died during the follow-up period. The mean survival time for all patients was 45.5 ± 19.1 months (range 12–80). Notch2 was overexpressed in 59 (51.3%) patients, although no significant relationship was identified with clinicopathological features such as age, gender, smoking, drinking, T stage, N stage and differentiation (Table 2). Kaplan-Meier analyses using the log-rank test were performed to calculate the effect of these clinicopathologic factors on the overall survival (OS) and progression-free survival (PFS) rates. The log-rank analysis demonstrated that high Notch2 protein expression significantly predicted decreased 5-year OS (29.1% vs. 49.1%; *P* = 0.013) and PFS (15.3% vs. 34.4%; *P* = 0.006) (Fig. 3A,B, Table 3). Furthermore, multivariate analysis identified Notch2 overexpression as an independent prognostic factor for OS (HR (95%CI): 2.266 (1.367–3.756), *P* = 0.002) as well as PFS (HR (95%CI): 2.160 (1.374–3.396), *P* = 0.006, Table 3). We also conducted receiver operating characteristic (ROC) analysis, and the area under the curve (AUC) value of Notch2 was 0.63 ± 0.06 (*P* = 0.044) according to the OS prediction and 0.61 ± 0.54 (*P* = 0.044) according to the PFS prediction (Fig. 3C,D).

### Notch2 inhibition with shRNA decreased EC-9706 cell proliferation and survival ability

**Figure 4:**
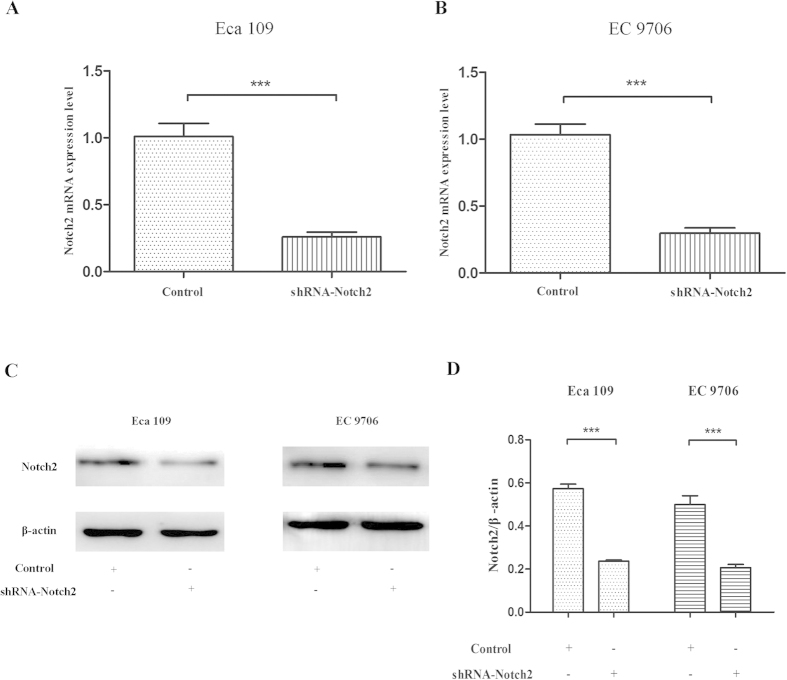
The mRNA and protein levels of Notch2 in shRNA-Notch2 and Control groups. (**A**) The mRNA level was decreased in Notch2-shRNA group via qRT-PCR. (**B**) The bands of Notch2 in western blot assay. (**C**) Quantitative analysis of Notch2 protein expression normalized to β-actin expression.

**Figure 5:**
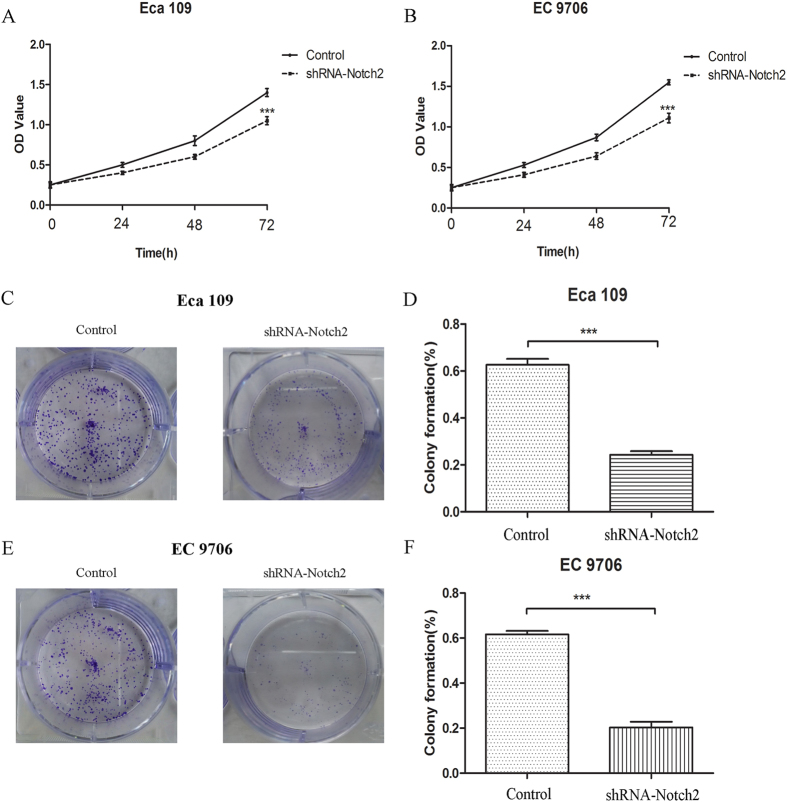
The results of CCK8 in Eca 109 (**A**) and EC 9706 (**B**) cell lines. The OD450 values were reduced in shRNA-Notch2 groups. The colongenic assay conducted in Eca 109 (**C**) and EC 9706 (**E**) showed that colony formation rates were decreased in shRNA-Notch2 group (**D,F**).

ESCC cell lines (Eca 109 and EC 9706) were firstly transfected with shRNA- Notch2. qRT-PCR and western blot were used to test the efficacy of transfection. As shown in Fig. 4A,B, Notch2 mRNA level in test group were significantly induced compared with the control group (Notch2/β-actin: 0.26 ± 0.04 vs 1.01 ± 0.10, *P* < 0.0001 and 0.30 ± 0.04 vs 1.03 ± 0.08, *P* < 0.0001, resp.). Fig 4C,D showed that the protein level of Notch2 was also decreased in transfection group (Notch2/β-actin: 0.24 ± 0.01 vs 0.57 ± 0.02, *P* < 0.0001, and 0.20 ± 0.02 vs 0.50 ± 0.04, *P* < 0.0001, resp.). To determine if knock-down of Notch2 expression by shRNA could lead to a decrease in the proliferation and survival of ESCC cells, CCK8 and clonogenic assay were conducted. The OD450 values of the Eca 109 and EC 9706 cells transfected with shRNA-Notch2 showed significant decrease at 24, 48, and 72 h (all *P* < 0.0001), compared with those cells in the control groups (Fig. 5A,B). The colony formations of transfected Eca 109 and EC 9706 were also significantly reduced compared with the control groups (all *P* < 0.0001, Fig. 5C,D).

## Discussion

Notch signalling has several demonstrated essential roles in the regulation of tumour growth, invasion, metastasis and angiogenesis^27^. Furthermore, the overexpression and oncogenic role of Notch2 have been observed in numerous human cancer types, such as lung adenocarcinoma^8^, glioma^9^, cervical cancer^10^,^11^, hepatoblastoma^12^, gastric cancer^13^,^14^ and salivary adenoid cystic carcinoma^15^. By contrast, there is evidence supporting a suppressive role for Notch2 in bladder cancer^16^, lung cancer^17^, breast cancer^19^ and colorectal cancer^20^. In the present study, we investigated the Notch2 expression level in ESCC tissues and analysed its prognostic value. For the first time, via qRT-PCR, IHC and western blot analyses, we found that Notch2 was up-regulated in frozen cancerous tissues compared with paracancerous tissues at the mRNA (*P* < 0.0001) and protein (IHC: *P* = 0.004; western blot: *P* = 0.021) levels. Similarly, Notch2 was overexpressed in 51.3% of FFPE tissues. Additionally, IHC staining revealed that Notch2 was primarily localized to the cytoplasm of cancer cells. These findings suggest an oncogene role for Notch2 in ESCC. However, Notch2 expression showed no significant relationship with clinicopathological features, such as the age, gender, smoking, drinking, T stage, N stage and differentiation, which may be due to the small quantity of sampling.

Notch2 has the potential to serve as a predictive biomarker in a variety of cancers. Accordingly, Notch2 mutations have been associated with a poor prognosis in splenic marginal zone lymphoma^6^, and the loss of Notch2 positively predicts survival in subgroups of patients with glial brain tumours^28^. A similar prognostic value was also demonstrated for Notch2 in liver cancer^29^. By contrast, a synergistic effect of positive Notch1 and negative Notch2 coexpression in predicting poor overall survival has been demonstrated^30^, and high Notch2 expression was shown to predict good survival for breast cancer patients^19^. In the present study, log-rank and multivariate analyses demonstrated that Notch2 expression in cancer tissues served as an independent prognostic factor for OS and PFS in ESCC patients. In particular, high Notch2 protein expression significantly predicted decreased 5-year OS and PFS; the T and N stages were also identified as prognostic indicators. The ROC-AUC was provided for the Cox regression models. It is a value that indicates the concordance level between observed and expected ordering of the data, and represents the percentage of concordance of all pairs of data with different outcome values, with concordance defined as occurring within a pair when the observation with the higher outcome value also has the higher predicted probability of the outcome. The AUC value for Notch2 was significant according to the OS and PFS predictions. Furthermore, CCK8 and clonogenic assay were conducted to determine if Notch2 inhibition could lead to a decrease in the proliferation and survival of ESCC cells. As expected, the results indicated that OD values and colony formations were significantly reduced in transfected group. Thus, we can conclude that Notch2 can be used as a biomarker for predicting ESCC survival in patients who underwent surgery. Besides, Notch1 has been widely reported in esophageal cancer^22^,^23^. It is also a novel potential prognostic biomarker for ESCC patients^31^,^32^. Further studies are necessary to investigate associations of Notch pathway signal molecules and synergistic effect of Notch1 and Notch2 coexpression in predicting survival of ESCC.

The detailed mechanisms of Notch2 have been studied and reported in several tumours. The silencing of Notch2 inhibits glioma cell proliferation by inducing cell cycle arrest and apoptosis *in vitro* and *in vivo*^9^,^33^. Constitutive Notch2 signalling in neural stem cells has been reported to promote tumorigenic features and astroglial lineage entry^34^. As a target gene of miR-107^35^, miR-204-5p^10^ and miR-23b^13^, Notch2 may also regulate cell migration and tumour invasion. In particular, Notch2 may negatively regulate cell invasion by inhibiting the PI3K-Akt signalling pathway in gastric cancer^36^. In salivary adenoid cystic carcinoma tissues, Notch2 may target HEY2 and CCND1 to regulate cell proliferation, invasion, and migration. Furthermore, the loss of the Notch pathway was shown to promote the epithelial-mesenchymal transition in bladder cancer cells, which was partially mediated by the loss of HES1^16^. Moreover, Notch2 activation by ZER inhibits its proapoptotic and anti-migratory response in breast cancer cells^37^. However, the mechanisms governing the role of Notch2 in ESCC have not been reported, and further studies are necessary to elucidate these cellular processes.

In conclusion, Notch2 is up-regulated in ESCC tissue compared to matched paracancerous tissue, and its overexpression could serve as a promising biomarker to identify individuals with poor prognostic potential.

## Methods

### Patient recruitment and data collection

Thirty pairs of ESCC and paracancerous tissue from patients were collected from October 2014 to March 2015 from Qilu Hospital of Shandong University. In addition, 140 FFPE cancer tissue samples from patients who underwent subtotal esophagectomy and esophagogastric anastomosis plus regional lymph node dissection in Qilu Hospital during 2009 were collected. Because 25 patients were lost to follow-up, we included 115 cases in the prognostic analysis. All cases were pathologically confirmed as ESCC. Patients did not receive chemotherapy or radiotherapy before surgery. We obtained the relevant data, including age, sex, smoking and drinking habits, histologic grade, invasion depth (T stage), lymph node metastasis (N stage), distant metastasis (M stage), differentiation degree and number of dissected lymph nodes, from clinical or pathologic records. The tumour, node, metastasis (TNM) classification was performed according to the American Joint Committee on Cancer staging manual (7th edition, 2010). The study protocol was approved by the ethics boards of Qilu Hospital, and tissue specimen acquisition was performed in accordance with the institutional guidelines. The written informed consent was obtained from all subjects.

### Culture of ESCC cell lines

Two established human ESCC cell lines (Eca109 and Eca9706) were used for this study. Both of the two cell lines were cultured in RPMI 1640 (Gibco BRL, Gaithersburg, MD) supplemented with 10% fetal bovine serum (FBS, Gibco), 100 U/ml penicillin G and streptomycin in a 37 °C incubator with humidified atmosphere and 5% CO_2_.

### Quantitative real-time polymerase chain reaction (qRT-PCR)

Surgical specimens were processed immediately after surgery. Total RNA was extracted from tissues using TRIzol reagent (Invitrogen, Carlsbad, USA) according to the manufacturer’s protocol. Complementary DNA (cDNA) was generated using qPCR RT Kit (Toyobo, Osaka, Japan) according to the manufacturer’s instructions. Primers were made by Sangon Biotech (Sangon Biotech, Shanghai, China). The primer sequences were as follows: Notch2 Forward primer: 5′-GGGACCCTGTCATACCCTCT-3′ and Reverse primer: 5′-GAGCCATGCTTACGCTTTCG-3′; β-actin Forward primer:5′-CAAAGGCCAACAGAGAGAAGAT-3′ and Reverse primer: 5′-TGAGACACACCATCACCAGAAT-3′. PCR was performed at 95 °C for 1 min and 40 cycles of 95 °C for 15 s, 58 °C for 15 s and 72 °C for 45 s. Notch2 expression was quantified using a Bio-Rad Single Color Real-Time PCR system (Bio-Rad, Hercules, California, USA) and calculated according to the mathematical model R = 2^−ΔΔCT^, where ΔCT = CT_Notch2_ − CT_β-actin_, and ΔΔCT = ΔCT_test_ −ΔCT_control_. All RT-PCRs were performed in triplicate, and the data are presented as the mean ± SD.

### IHC staining

All fresh specimens were collected during surgery, fixed with 10% formalin and embedded in paraffin. The FFPE cancerous tissues from 2009 were collected from the Department of Pathology of Qilu Hospital. These tissues were cut as 4-mm serial sections. Following deparaffinization with xylene and rehydration, the sections were retrieved in 10 mM citrate buffer. Then, incubation in 3% H_2_O_2_ in methanol for 20 min at room temperature was used to block the endogenous peroxidase enzyme activity. The slides were then incubated with primary rabbit anti-Notch2 polyclonal antibody (1:100, Abcam, Cambridge, MA, USA) overnight at 4 °C in a high humidity chamber, followed by incubation for 30 min at 37 °C with biotinylated secondary antibodies and streptavidin-peroxidase complex. Finally, a 3,3′-diaminobenzidine solution was added, and the slides were counterstained with haematoxylin and mounted with neutral balsam. For negative controls, sections were incubated with PBS instead of the primary antibodies.

The sections were observed under a light microscope and independently scored by three investigators. Conflicting scores were resolved by selecting the value that was consistent between two observers or the average of the scores. The final score was determined by multiplying the staining intensity (scored as: 0, no staining; 1, weak staining; 2, moderate staining and 3, strong staining) by the percentage of positive cells (scored as: 0, 0–10% positive cells; 1, 10–25% positive cells; 2, 26–50% positive cells; 3, 51–75% positive cells; and 4, 76–100% positive cells). The final staining score was the sum of the staining intensity and percentage of positive cells, and it was further graded as follows: 0–1, (−); 2–3, (+); 4–5, (++); and 6–7, (+++). The expression of Notch2 was divided into a non-overexpressed group (− or +) and an overexpressed group (++ or +++).

### Protein extraction and western blot

Protein was extracted from tissues. Tissues were pestled in liquid nitrogen and added to RIPA Lysis Buffer (50 mM Tris, 150 mM NaCl, 1% Triton X-100, 1% sodium deoxycholate, 0.1% SDS, sodium orthovanadate, sodium fluoride, EDTA, leupeptin) and PMSF (Phenylmethanesulfonyl fluoride, Beyotime,China), that the ratio is 100:1. Protein extracts were electrophoresed on 10% SDS polyacrylamide gels and transferred to nitrocellulose membranes. Membranes were blocked with 5% non-fat dried milk and incubated overnight with an appropriate primary Notch2 antibody (1:1,000, Abcam, Cambridge, MA, USA) and β-actin antibody (1:1,000, Abcam, Cambridge, MA, USA). Then, horseradish peroxidase-conjugated secondary antibodies were added. Bands were subsequently visualized using a chemiluminescence detection system (EMD Millipore, Billerica, MA, USA) and density was determined using an image analyzer. β-actin was used to ensure equivalent protein loading.

### Transfection

The Eca 109 and EC 9706 cells were transfected with specific Notch2 shRNA (Genechem, Shanghai, China) for 72 h using Lipofectamine™ reagent in serum-free 1640 medium according to the manufacture’s instruction. The multiplicity of infection was 20 for Eca 109, and 40 for EC 9706 cells. Untreated cells were used as a negative control. The efficacy of transfection was tested by qRT-PCR and western blot.

### CCK8 assay

The Eca 109 and EC 9706 cells proliferation was detected using 2-(4-indophenyl)-3-(4-nitrophenyl)-5-(2,4-disulphophenyl)-2 Htetrazolium monosodium salt (cell counting kit-8 (CCK8)). After transfection, the logarithmically growing cells were seeded in 96-well plates at a cell density of 5 × 10^4^/well and incubated for 0, 24, 48, and 72 h. At different time intervals, the cells were incubated with CCK8 reagent for 1 h at 37 °C. The absorbance of each well was measured at 450 nm using Thermo Scientific Varioskan Flash (Thermo Scientific, Finland). Percentage of viable cells = (OD450 of treated sample −OD450 of blank sample)/(OD450 of control sample −OD450 of blank sample) ×100%. The results shown were mean values of three independent experiments.

### Clonogenic assay

Clonogenic assay was also used to evaluate the function of Notch2. Transfected cells were trypsinized to generate a single cell suspension and seeded in 6-well plates at 500 cells per well. 14 days after seeding, colonies were stained with crystal violet, and the number of colonies containing at least 50 cells was counted. The colony survival fraction was calculated for each treatment.

### Statistical analysis

The difference in the Notch2 mRNA and protein level between cancerous and paired paracancerous tissues, as well as difference in Notch2 level, OD450 value and colony formation between shRNA-Notch2 and control group was compared using a paired Student’s t-test. The chi-square test was used to test the correlation between Notch2 expression and clinicopathological factors. The Kaplan–Meier method was used to calculate the survival curves, and the log-rank test was used to compare the survival difference between patient subgroups. Multivariate Cox regression analysis was used to identify significant independent prognostic factors. The ROC-AUC was also provided for the Cox regression models. AUC values are calculated for each of the adjusted models to illustrate the predictive ability of the independent variables. Differences between groups were considered significant for *P* values < 0.05. All statistical analyses were performed with SPSS 17.0 statistical software (SPSS Inc., Chicago, IL, USA).

## Additional Information

**How to cite this article**: Wang, C. *et al*. Notch2 as a promising prognostic biomarker for oesophageal squamous cell carcinoma. *Sci. Rep.* **6**, 25722; doi: 10.1038/srep25722 (2016).

## Figures and Tables

**Table 1:** Quantification of the expression of Notch2 in cancerous and paracancerous tissues via IHC staining.

Group	n	Overexpression (n)	Overexpression rate (%)	χ^2^	*P*value
Cancerous tissue	30	19	63.3%	8.148	0.004
Paracancerous tissue	30	8	26.7%		

Abbreviation: IHC, immunohistochemical.

**Table 2:** The correlation of ESCC clinicopathologic variables with Notch2 expression in FFPE cancerous tissues.

Clinicopathological features	Notch2 overexpression		*P*^a^ value
	No (n = 56)	Yes (n = 59)	
Age			0.530
<65	30	31	
≥65	26	28	
Gender			0.454
Female	25	28	
Male	31	31	
Smoking			0.381
No	45	45	
Yes	11	14	
Drinking			0.209
No	40	47	
Yes	16	12	
Differentiation			0.066
Well	27	21	
Moderate	10	22	
Poor	19	16	
T stage			0.363
T1	10	5	
T2	17	25	
T3	20	19	
T4	9	10	
N stage			0.323
N0	23	24	
N1	11	16	
N2	11	14	
N3	11	5	
TNM stage			0.238
I	19	15	
II	9	17	
III	28	27	

*P*^a^: Chi-square test. Abbreviation: FFPE, formalin-fixed paraffin-embedded.

**Table 3:** Univariate and multivariate analyses of prognostic variables.

Variable	OS Univariate analysis	OS Multivariate analysis		PFS Univariate analysis	PFS Multivariate analysis	
	*P* value	HR (95% CI)	*P* value	*P* value	HR (95% CI)	*P* value
Gender (Male vs. Female)	0.880	0.796 (0.494–1.281)	0.347	0.807	0.816 (0.530–1.257)	0.357
Age (<65 vs. ≥65)	0.656	0.839 (0.504–1.396)	0.498	0.676	0.892 (0.565–1.407)	0.622
Smoking (Yes vs. No)	0.497	1.226 (0.655–2.292)	0.524	0.922	0.994 (0.557–1.774)	0.985
Drinking (Yes vs. No)	0.105	1.222 (0.643–2.324)	0.541	0.134	1.230 (0.682–2.217)	0.491
T stage (T3 and T4 vs. T1 and T2)	<0001*	1.430 (1.094–1.869)	0.009*	0.002	1.489 (1.154–1.922)	0.002*
N stage (N2 and N3 vs. N0 and N1)	0.001*	1.442 (1.122–1.853)	0.004*	0.001*	1.308 (1.042–1.643)	0.021*
Differentiation (Well vs. Moderate and Poor)	0.079	0.881 (0.655–1.184)	0.402	0.100	0.946 (0.725–1.235)	0.682
Notch2 (Overpression vs. nonexpression)	0.013*	2.266 (1.367–3.756)	0.002*	0.006*	2.160 (1.374–3.396)	0.006*

Abbreviations: OS, overall survival; PFS, progression-free survival; CI: confidence interval.
